# Maxillary sinus lift response to platelet-rich plasma associated with autogenous bone, ceramic biphasic HA/β-TCP (70:30), or deproteinized bovine bone

**DOI:** 10.1186/s40729-020-00277-9

**Published:** 2020-11-30

**Authors:** Caroline Andrade Rocha, Ricardo Vinicius Nunes Arantes, Tania Mary Cestari, Paula Sanches Santos, Gerson Francisco Assis, Rumio Taga

**Affiliations:** Laboratory of Histology of Department of Biological Sciences, Bauru Dental School, University of São Paulo, Alameda Octávio Pinheiro Brisolla 9-75, Bauru, SP 17012-901 Brazil

**Keywords:** Platelet-rich plasma, Bone regeneration, Maxillary sinus

## Abstract

**Background:**

This study evaluated the long-term effects of platelet-rich plasma (PRP) on bone formation and regeneration when associated with autogenous bone graft (AB), porous biphasic calcium phosphate (pBCP), or deproteinized bovine bone (DBB) in maxillary sinus augmentation (MSA) of rabbit.

**Methods:**

In 54 rabbits, bilateral MSA procedure was performed and randomly one sinus was filled with 200 mm^3^ material plus blood clot (AB/clot, DBB/clot, and pBCP/clot) and other with the same graft plus PRP (AB/PRP, DBB/PRP, and pBCP/PRP). After 30, 60, and 180 days, microtomographic were performed to analyze the three-dimensional MSA volume and histomorphometric analyses for *the percentage* of bone and soft tissues ingrowth. Data were compared by two-way ANOVA and the means were compared by the Tukey test, at *p* < 0.05.

**Results:**

The percentage of pBCP and DBB were nearly unchanged throughout the whole period and bone formation occurred in the spaces between particles. The MSA volume filled with DBB and pBCP agglutinated with clot and PRP maintained constant during all experimental periods (147.2 mm^3^ and 154.9 mm^3^, respectively, *p* = 0.7377), and no significant changes in the new formatted bone and soft tissue were observed between treatments. In AB/clot and AB/PRP, the MSA volume was similar at 30 days (140.3 mm^3^ and 137.9 mm^3^, respectively), but a higher and gradual reduction was observed until 180 days. In the AB/PRP, this reduction was significantly higher (44.2%) than AB/clot (22.5%) (*p* = 0.01792). Histologically, the addition of PRP to AB accelerated the new bone formation/remodeling maintaining the percentage of new bone similar to AB/clot during all experimental volume (*p* = 0.6406), while the AB particles showed a higher resorption in AB/PRP than AB/clot until 60 days (mean of 7.8% and 15.1%, respectively, *p* = 0.0396).

**Conclusion:**

The association of PRP with the autogenous graft accelerates the process of bone formation/remodeling in MSA, but not had influence on the pBCP and DBB groups.

## Introduction

Alveolar bone resorption and pneumatization of the maxillary sinus after posterior tooth extraction often lead to low quality and unsuitable quantity of bone in the posterior maxilla, which makes it difficulty in the local rehabilitation with use of dental implants [1]. Maxillary sinus lift is the most widespread treatment to increase the height of residual bone between the crest and sinus floor to allow the placement of endosseous implants [2]. Autogenous bone (AB) is generally considered the gold standard graft for maxillary sinus bone augmentation because of their osteogenic, osteoinductive, osteoconductive, and non-immunogenic properties [3, 4]. However, factors such as donor site morbidity, limited availability of bone tissue, and unpredictable graft resorption are the major disadvantages for the use of autogenous bone [5]. As an alternative to autogenous bone, numerous grafting materials can be used as substitutes: allogenic or xenogenic bone matrix, alloplastic material, and a combination of various materials.

A good alternative to autogenous bone are graft materials consisting of deproteinized bovine bone (DBB) which has a microporous structure similar to that human cancellous bone and a higher osteoconductive properties with very low resorbability [6]. Calcium phosphates materials such as hydroxyapatite (HA), beta-tricalcium phosphate (ß-TCP) or biphasic calcium phosphate (BCP), the latter a mixture of HA and β-TCP, are also used as bone substitute materials for dental and orthopedic procedures. The combination of less soluble HA and highly resorptive β-TCP might be more effective in the balance between calcium phosphate degradation and bone formation [7].

Strategies to accelerate bone healing have included the use of platelet-rich plasma (PRP) that is a highly concentrated form of blood autogenous platelets that is rich in various groups of growth factors such as platelet-derived growth factor (PDGF), transforming growth factor-beta (TGF-β), vascular endothelial growth factor (VEGF), insulin growth factor 1 (IGF-1) and others [8]. Thus, PRP has been proposed as a complement to autogenous bone graft and osteoconductive grafting materials to improve bone healing [9]. The use of PRP in combination with different bone substitute materials for sinus lift has been analyzed in recent years. Some studies have reported positive effects [10], while others have shown limited effects relative to the efficacy of PRP in the increase of bone formation after sinus lift [11].

In the present work was used the experimental model of maxillary sinus lifting in rabbits. This animal species has a maxillary sinus with a well-defined ostium opening to their nasal cavities, similarly to humans [12]. So, this model is useful to verify the new bone formation after sinus lift because the space filler material is subjected to the air pressure against sinus membrane, similarly what occurs in human maxillary sinus, which may affect the increase and structure of new bone [13]. Besides that, the rabbit is appropriate for PRP investigations because rabbit’s hematological picture is similar to the human one [14].

Thus, the aim of this investigation was to evaluate histologically, histomorphometrically and tomographically the effect of PRP on bone formation and regeneration when associated with AB, DBB, or BCP in rabbit’s maxillary sinus lift model. We hypothesized that the use of PRP with these biomaterials results in an increased bone volume and/or improved bone structure in maxillary sinus augmentation (MSA) compared to blood clot.

## Material and methods

*Grafting materials:* porous biphasic calcium phosphate (pBCP): granules composed by hydroxyapatite and β-tricalcium phosphate at 70:30 ratio produced by Baumer S.A., Mogi Mirim, SP, Brasil, 0.5–0.75 mm particle size. Deproteinized bovine bone (DBB): Bio-Oss®, Geistlich Pharma AG, Wolhusen, Switzerland, 0.25–1 mm particle size. Autogenous bone (AB): particulate bone obtained of the two pieces of parietal bone surgically removed with an 8-mm trephine drill.

### Study design (Fig. 1a)

**Fig. 1:**
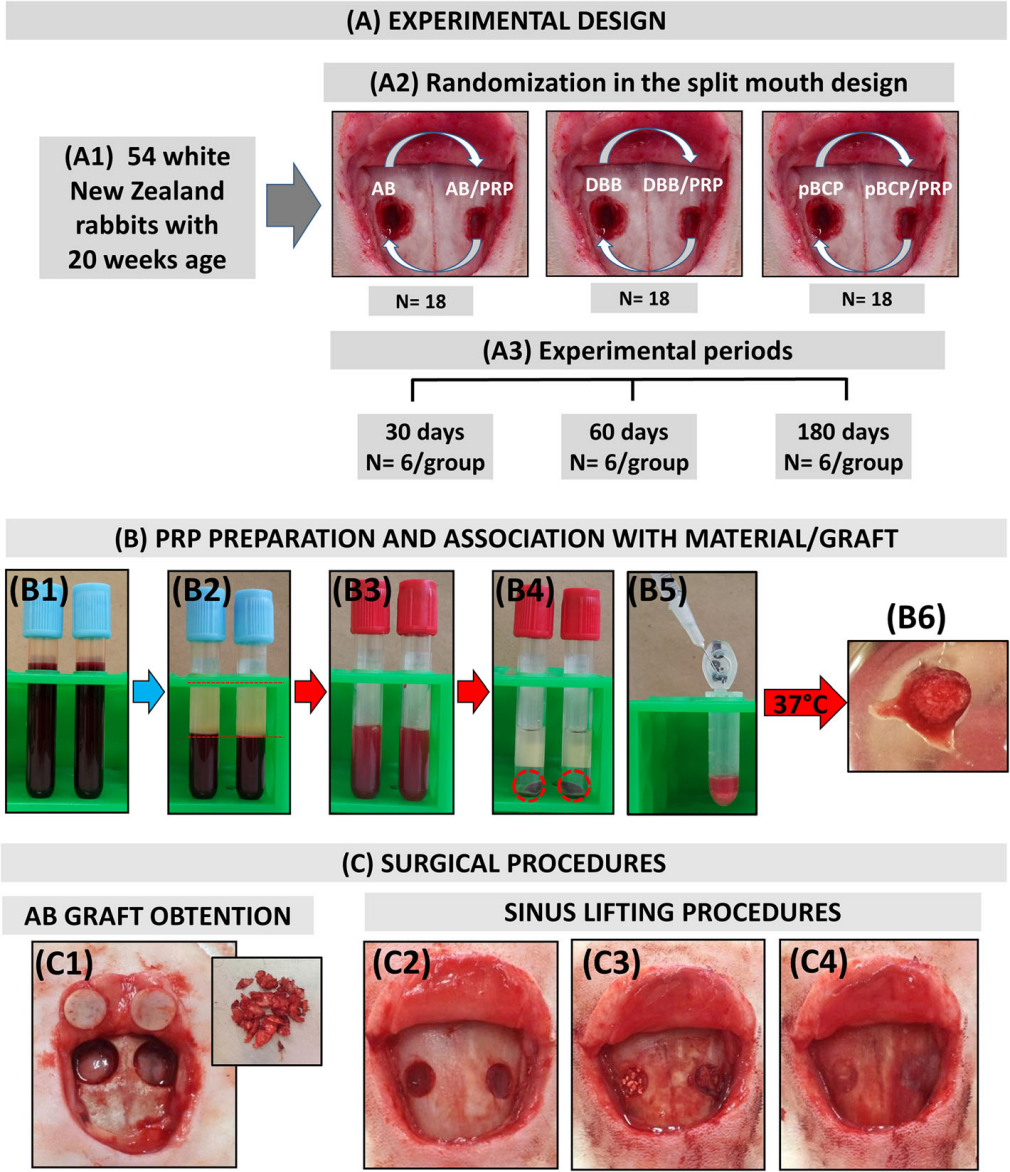
Materials and methods**. a** Experimental design: scheme shows animal data and the randomization of MSA treatment with different materials homogeneity with clot or PRP. **b** PRP preparation and association with material/graft: (**B1**) whole blood collected in two sterile glass tubes containing 3.8% sodium citrate. (**B2**) PPP/PRP separated from red blood cells after centrifuge at 200×*g* (1200 rpm) for 10 min. (B3) PPP/PRP separated and transferred to a tube without anticoagulant. (**B4**) After centrifugation at 200×*g* for 15 min note the separation of PPP and platelet plug (0.5 mL). (**B5**) PRP added to a sterile tube containing the space filler plus 0.06 mL of 10% CaCl solution. (**B6**) Tube immediately transferred to water bath at 37 °C for 10 min until gelation. **c** Surgical procedures: (**C1**) AB grafts obtained by an 8-mm trephine bur. (**C2**) The maxillary sinus opened bilaterally using a 4-mm trephine bur. (**C3**) Space fillers inserted into both sinuses. (**C4**) Bone windows covered with collagen membrane before suturing

Fifty-four adult male white New Zealand rabbits (3.97 ± 0.300 kg) with 20 weeks of age were used. The protocol of the experiment was approved by the Institutional Animal Care Committee (protocol 39/2011 of University of São Paulo, USP, Bauru, SP, Brazil) in accordance with the National Institutes of Health Guide for the Care and Use of Laboratory animals (NIH Publications No. 8023, revised 1978). In this study, ARRIVE guidelines for the reporting of animal studies was followed [15]. The rabbits were divided in three experimental groups according to graft materials AB (*n* = 18), DBB (*n* = 18), and pBCP (*n* = 18). In each rabbit, bilateral MSA procedure was performed. Randomly one sinus was filled with graft materials plus blood clot and other with the same graft plus PRP according to a split-mouth design (Fig. 1 A2). The animals of each group were assessed at three distinct time points: 30 (*n* = 6/group), 60 (*n* = 6/group), and 180 (*n* = 6/group) days (Fig. 1 A3). Each animal was placed in individual cages, receiving a regular diet and allowing unrestricted physical activity.

### Preparation of autologous PRP

On the surgery day, the animal was anesthetized and approximately 11 mL of whole blood was drawn via cardiac puncture. Blood was collected in two sterile glass tubes containing 3.8% sodium citrate (BD Vacumtainer Ref. 367841) and centrifuged using a two-step procedure [16]. The initial centrifugation was done at 200×*g* (1200 rpm) for 10 min to separate platelet-containing plasma from the red and white blood cells (Fig. 1 B1–B2). All plasma along with the top erythrocyte layer was collected using a micropipette (Yale spinal needle, Becton and Dickinson, USA) and transferred to an empty vacutainer tube (BD Vacumtainer Ref 367812) without anticoagulant and centrifuged at 200×*g* (1200 rpm) for 15 min (Fig. 1 B3–B4). Through this second centrifugation, platelet-poor plasma (PPP) was separated from PRP. After the removal of PPP, the volume of PRP of the approximately 0.5 mL was collected. The platelets present in the whole blood and in PRP were counted automatically using a hematological analyzer (Pentra 80, Horiba-Abx, Japan). Therefore, after placement of 0.5 mL of PRP in a sterile tube containing the space filler, it was added 0.06 mL of 10% calcium chloride solution for activation (Fig. 1 B5). The tube was then transferred immediately to water bath at 37 °C for 10 min until gelation.

### Surgical procedures

One experienced surgeon performed all surgeries in one surgical center under strict sterile conditions from the morning. Each rabbit was anesthetized by intramuscular injection of 10 mg/kg xylazine hydrochloride (Anasedan®, Ceva Saúde Animal Ltda, Paulínea, SP, Brazil) and 45 mg/kg ketamine hydrochloride (Dopalen®, Ceva Saúde Animal Ltda, Paulínea, SP, Brazil). When necessary, 1/3 of the original calculated dose of ketamine was administered. Only in AB group, a second surgical area (donor area) was needed. The autogenous calvarial bone graft was harvested from the parietal bones (see Fig. 1c). In brief, a half-moon incision on the calvarium skin was made and the flap raised posteriorly exposing the *parietal bone surfaces*. Using an 8-mm-diameter trephine bur under continuous saline irrigation, two pieces of autogenous bone was removed and crushed (Fig. 1c). Next, on all rabbits, the MSA procedures were realized in according to Xu et al. (2003) [17]. Briefly, a half-moon incision was made to expose the nasal bone and the naso-incisal suture lines. Using a 4-mm-diameter trephine bur under continuous saline irrigation, two circular windows were created bilaterally in the nasal bones of each rabbit, exposing the Schneiderian membrane that was elevated and the cavity between the mucosa and the inferior osseous septum of the sinuses was augmented with 200 mm^3^ of graft particles plus blood or PRP (Fig. 1 C2–C3). A resorbable collagen membrane (GenDerm, Baumer SA, Mogi Mirin, SP, Brazil) was placed above window (Fig. 1 C4) and the periosteum and the skin were closed with 4-0 black silk line (Ethicon®-Johnson and Johnson®, Brazil).

### Postoperatory procedures

The postoperative care consisted of subcutaneous injection of 10 mg/kg of enrofloxacin 2.5% (Flotril® 2.5%; Intervet-Schering-Plough, Brazil) for 7 days and 0.1 mL/kg of anti-inflammatory ketoprofen (Ketofen 1%, Merial, Brazil) for 3 days.

### Sample collection and X-ray microtomography analysis

At 30, 60, and 180 days after surgery, each rabbit was anaesthetized with xylazine/ketamine and posteriorly, euthanized by intracardiac injection 30 mg/kg (90–110 mg/kg) of sodium pentobarbitone (Nembutal®). The maxillary sinuses were collected, fixed in 10% buffered formalin for 10 days and scanned using a Skyscan 1176 high-resolution desk-top micro-CT system (Skyscan®, Kontich, Belgium) operated at 80 kV and 300 μA, using a 0.5-mm filter of Cu. For each maxillary sinus, a total of 394 scan slices at a resolution of 17.5 μm at rotation range of 180° with a rotation step of 0.5° were obtained. The corresponding 3D reconstruction images were carried out by using software NRecon 1.6.1.5 (Skyscan®, Kontich, Belgium). Analysis of the 3D images allowed the calculation of the total volume of the regenerated space (TV) at 30, 60, and at 180 days.

### Histological procedures and histomorphometry

After micro-CT scanning, the samples were processed by decalcification in 4.13% ethylenediaminetetraacetic acid (Titriplex III-Merck KGaA, Damstadt, Germany) plus 0.44% sodium hydroxide for approximately 60 days and inclusion in polymer-enriched paraffin (Histosec–Merck). Coronal semiserial 5-μm-thick sections were obtained along the center of the augmented sinus and stained with hematoxilin and eosin (HE) for the light microscopic examination (BX50, Olympus Co., Tokyo, Japan). The identity of the histological sections was blinded during analysis to avoid evaluation bias.

Histomorphometric analysis were performed by a single calibrated investigator in a Zeiss Axioscope light microscope (Carl Zeiss Microscopy GmbH, Jena, Germany) equipped with a × 20 objective and an × 10 Kpl eyepiece containing an integration test grid of 100 points. The manual point-counting method (Weibel; 1969) was used for determination of *percentage* of each structure (new bone, materials, connective tissue and bone marrow) in each augmented sinus. Thus, the grid was overlaid in 20 histological fields per maxillary sinus selected by systematic sampling (Weibel; 1969). For each maxillary sinus augmented, the percentages of new bone (NB%), connective tissue (CT%), materials (M%), and bone marrow (BM%) was calculated by relation pi/P, where pi is the number of points over a determined component, and *P* is the total number of points over the entire examined area.

### Statistical analysis of data

The microtomographic and histomorphometric data were checked for normal distribution and homogeneity of variance by test of Hartley, Cochran, and Bartlett and were compared by two-way analysis of variance (ANOVA), allowing a test for interaction between the two factors (time and treatment). Finally, the post hoc Tukey test was carried out in order to evaluate the “individual” effect on each examined variable. Paired *t* test was used to evaluate differences between the initial and final weight of the animals and the number of platelets was analyzed using the one-way ANOVA. Two-way ANOVA was used to assess the existence of interaction between graft materials and treatment. The software Statistica (Version 10.0; StatSoft Inc., Tulsa, OK, USA) was used for the data analysis, considering the level of significance of 5% (*p* < 0.05).

## Results

All animals tolerated the surgical procedures and remained healthy throughout the experimental period without the occurrence of any adversities. In Table 1, the total number of platelets in whole blood (NP-B), autologous PRP and platelet-poor plasma (NP-PPP) is shown. The PPP is blood plasma with very low number of platelets (< 10 × 10^3^/μL) use in platelet aggregation studies to both adjust the platelet-rich plasma concentration and to serve as a control [18]. In our study, the NB-PPP was in average of 6.2 ± 4.21 × 10^3^/μL per μL, showing a good standardization of the technique for obtaining the PRP. The total number of platelets in the whole blood (NP-B) and PRP were similar among AB, DBB, and pBCP groups. But, in all treatment groups, the total number of platelets in PRP was 419.5% higher compared to whole blood (average of NP-PRP of 1403 ± 384.1 × 10^3^/μL vs. NP-B of 270.3 ± 51.83 × 10^3^/μL, *p* < 0001).

**Table 1:** Mean and ± SD (minimum and maximum) number of Platelets in whole blood (NP-B), PRP (NP-PRP), PPP (NP-PPP), and PRP platelet increase. One-way ANOVA

Group	NP-B (× 10^**3**^/μL)	NP-PRP (× 10^**3**^/μL)	Paired “***t***” test (***p*** values)	Platelet increase in PRP (%)	NP-PPP (× 10^**3**^/μL)
**AB (*n*** **= 18)**	265.3 ± 56.81 (159–361)	1357 ± 439.1 (792–2237)	< 0.0001	411.9 ± 103.68 (174-621)	6.5 ± 4.42 (0–19)
**DBB (*n*** **= 18)**	269.4 ± 35.11 (217–348)	1453 ± 279.8 (761–2171)	< 0.0001	438.4 ± 90.77 (232–608)	5.1 ± 3.60 (0–14)
**pBCP (*n*** **= 18)**	276.2 ± 63.57 (159–389)	1401 ± 433.6 (792–2237)	< 0.0001	408.4 ± 98.50 (174–621)	7.1 ± 4.63 (0–19)
**ANOVA “*p*"**	0.8258	0.7625		0.6043	0.1669^a^

^a^Kruskal-Wallis test

**Table 2:** Mean and standard deviation obtained for MSA volume and percentages of new bone (NB%), connective tissue (CT%), materials (M%), and bone marrow (BM%) for all groups and experimental periods

Parameter	Periods	AB		DBB		pBCP	
		AB/clot	AB/PRP	DBB/clot	DBB/PRP	pBCP/clot	pBCP/PRP
MSA volume (mm^3^)	30	140.33 (17.26)	137.98 (13.41)	149.83 (10.51)	135.06 (21.44)	143.2 (15.75)	145.4 (20.34)
	60	131.91 (25.37)	105.84 (12.62)	148.14 (33.55)	144.69 (22.49)	164.47 (13.86)	153.27 (18.26)
	180	108.69 (31.85)	76.39 (25.41)	151.16 (24.13)	154.25 (34.48)	160.08 (11.18)	159.15 (13.33)
M%	30	17.35 (7.94)	7.91 (5.28)	42.33 (5.07)	44.71 (2.27)	46.81 (3.00)	44.43 (8.99)
	60	12.80 (5.93)	7.65 (3.32)	34.99 (3.73)	29.44 (4.21)	40.25 (2.85)	33.73 (4.03)
	180	2.27 (4.21)	4.51 (2.21)	30.92 (2.36)	30.08 (3.51)	37.03 (3.21)	38.66 (2.21)
CT%	30	12.45 (6.96)	16.01 (5.11)	29.92 (4.89)	31.66 (6.31)	20.38 (6.58)	26.56 (5.78)
	60	7.73 (7.05)	7.57 (3.00)	16.47 (5.01)	19.00 (5.97)	14.48 (2.62)	15.92 (2.47)
	180	7.76 (2.75)	8.02 (1.09)	9.23 (1.67)	11.01 (2.55)	14.25 (1.80)	11.38 (3.58)
NB%	30	30.00 (2.81)	34.43 (6.27)	22.46 (4.63)	21.21 (6.80)	21.96 (4.49)	19.49 (3.77)
	60	36.78 (6.17)	29.60 (5.38)	25.99 (0.87)	26.63 (1.64)	27.65 (1.08)	27.31 (2.33)
	180	18.79 (4.69)	24.09 (2.71)	27.11 (2.27)	28.55 (2.65)	25.20 (2.10)	27.06 (1.88)
BM%	30	39.95 (13.66)	41.54 (5.73)	4.74 (3.65)	1.84 (1.41)	10.09 (5.01)	8.50 (9.44)
	60	42.44 (10.40)	55.11 (5.78)	22.22 (4.83)	24.26 (6.04)	17.75 (2.97)	22.64 (5.72)
	180	71.04 (8.13)	63.34 (5.27)	32.52 (5.50)	30.08 (4.15)	22.55 (3.41)	22.21 (5.09)

### Morphologic and volumetric microCT analysis: 3D volume variation

**Fig. 2:**
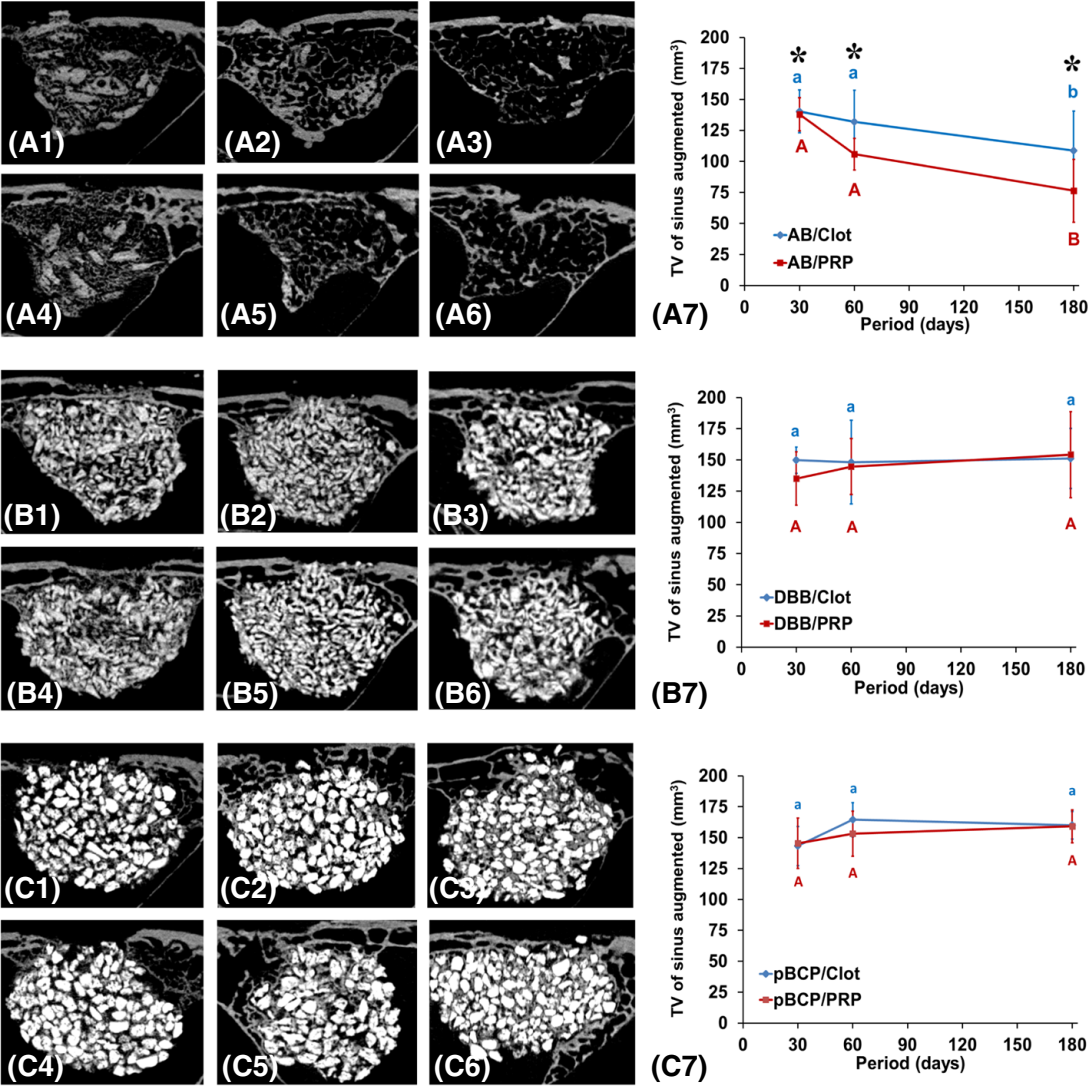
Micro-CT evaluation. **a** 2D microCT–panoramic view: sagittal images show in AB treatment at 30 days large AB particles with the presence of fine bone trabeculae being more evident in the AB/PRP group than AB/clot At 60 and 180 days gradual reduction of AB particles with formation of thicker but less frequent bone trabeculae. In DBB and pBCP with clot and PRP show particles hyperdense than remaining bone tissue. Between 30 and 180 days, an increase of isodense areas in the space between particles referent to new bone tissue is shown. **b** TV of sinus augmented: graphs of evolution of MSA volume obtained by AB/clot and AB/PRP (**A7B1**), DBB/clot and DBB/PRP (**B7B2**), and pBCP/clot and pBCP/PRP (**C7B3**). Different letters *p* < 0.05 among groups and periods

The panoramic view of MSA with different graft materials in each experimental period and graphic of total volume (TV) of sinus augmented are presented in the Fig. 2 and numerical data in the Table 2. In AB/clot and AB/PRP subgroups (Fig. 2 A1, A4), the MSAs were filled with isodense and large AB particles interspersed with thin bony trabeculae at 30 days. In this period, TV of MSA (Fig. 2 A7) filled with AB/PRP was similar to AB/clot being in mean of 139.2 ± 15.33 mm^3^. At 60 days, a higher reduction of AB particles occurred in the AB/PRP relative to AB/clot leading a higher reduction of MSA volume (TV of 105.8 mm^3^ vs. 131.9 mm^3^). In this period, both groups showed reduction on the number of trabeculae in newly formed cancellous bone and an increase in their thickness (compare the Fig. 2 A1; A4 with A2; A5). At 180 days, in both AB treatments, the MSA was filled by fine trabecular bone and rare fragments of AB particles with reduction of MSA-TV (mean of 76.4 mm^3^ for AB/PRP and 108.7 mm^3^ for AB/clot). In DBB and pBCP groups plus clot or PRP, the MSA was filled with large amount of hyperdense particles of DBB (Fig. 2 B1–B6) and pBCP (Fig. 2C1–C6) during all experimental periods. An increase of isodense images referring to new bone formation was present in the space between particles. Relative to MSA-TV, in both groups no statistical differences occurred between treatments with clot or PRP (Fig. 2 B7 and C7).

### Descriptive and histomorphometric analysis

#### Clot vs. PRP per each graft material

**Fig. 3:**
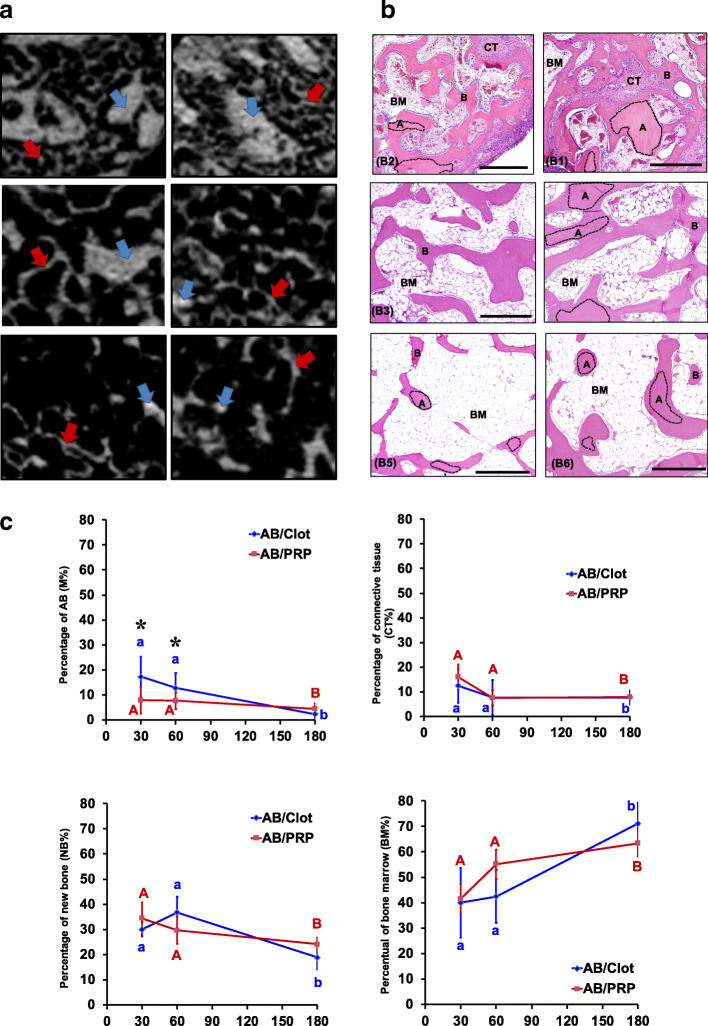
MicroCT and histomorphometric results for AB group. **a** 2D-microCT view: the images show large AB particles (blue arrow) and many fine trabeculae of new bone (yellow arrow) between particles at 30 days. Between 30 and 180 days, reduction of AB particles (blue arrow) and thickening and reduction of the number of bone trabeculae occur. **b** Histological view at 30 days, AB particles (area surrounded by black dotted line) are surrounded by bone (yellow arrow) and the spaces between them filled by connective tissue (CT) and bone marrow (BM). Note that at 60 and 180 days, the new formatted bone and AB particles reduces and most of the space between the particles was replaced by bone marrow (BM). HE; 10, **c**) Percentage of each constituent: graphs of evolution of M% (**C1**), CT% (**C2**), NB% (**C3**), and BM% (**C4**) in MSA of AB/clot and AB/PRP. Different letters *p* < 0.05 among periods per group and asterisks difference between groups

AB group (Fig. 3 and Table 2): Micro-CT and histological views at 30 days showed large and irregular AB particles surrounded by immature newly formed bone tissue composed of fine trabeculae (Fig. 3 A and B1, B2). Although, the initial volume of the particles of AB plus clot or PRP grafted were similar (200 mm^3^), the M% was significantly smaller in AB/PRP (7.9%) than AB/clot (17.3%) at 30 days (*p* = 0.0395) (Fig. 3 C1). However, no differences occurred in the percentage of new trabecular bone. Between 30 and 180 days, the M% decreased 14% in AB/clot and 56% in AB/PRP, occupying only 2.3% and 4.5% of MSA, respectively (Fig. 3 C1). Concomitantly, the NB% (Fig. 3 C3) in AB/clot and AB/PRP reduced 63% (30% to 18%) and 71% (34.4% to 24.1%), respectively, while the BM% (Fig. 3 C4) increased 76% (39.9% to 70.6%) and 52% (41.5% to 63.3%), respectively. In this case, no differences were observed between treatments (*p* = 0.6406 and 0.46884, respectively).

**Fig. 4:**
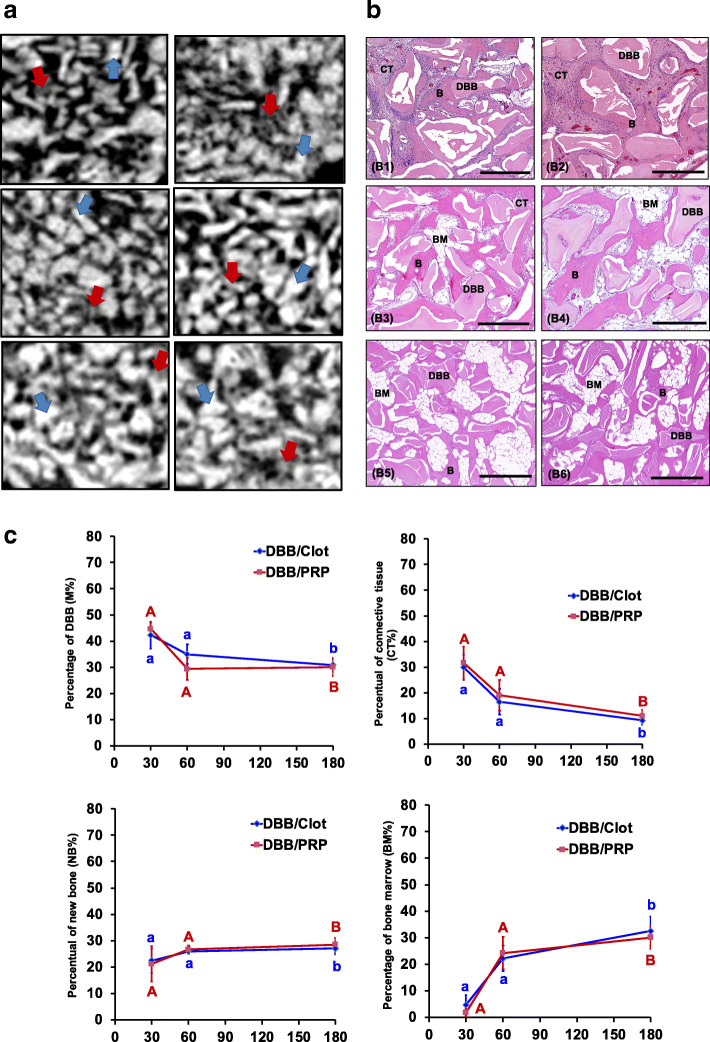
Micro-CT and histomorphometric results for DBB group. **a** 2D-Micro-CT view: the images show small and irregular DBB particles (blue arrow) surrounded by new bone (yellow arrow) at 30 days. Between 30 and 180 days, the spaces were filled by bone. **b** Histologic view: at 30 days note that the bone formation occurred on the surface of the DBB (area surrounded by black dotted line), the spaces were filled by connective tissue (CT) rich in cells and blood vessels. At 60 days, the spaces between the particles were occupied by a large amount of bone (yellow arrow) and bone marrow (BM) replacing connective tissue. At 180 days, lamellar bone tissue and some areas of osteoclastic reabsorption in both biomaterial and bone. HE; 10×, **c**) Percentage of each constituent: graphs of evolution of M% (**C1**), CT% (**C2**), NB% (**C3**), and BM% (**C4**) in MSA of DBB/clot and DBB/PRP. Different letters *p* < 0.05 among periods per group

DBB group (Fig. 4 and Table 2): Micro-CT and histological views at 30 days showed higher quantity of DBB particles in both subgroups (M% = 42.3% and 44.7% for DBB/clot and DBB/PRP, respectively, Fig. 4 C1) with small size and irregular format in the MSA (Fig. 4a, B1, B2, and C1). A thin layer of newly formed bone was present on the DBB surface occupying 22.4% of MSA in DBB/clot and 21.2% in DBB/PRP (Fig. 4 B1, B2, and C3). The space between DBB/new bone was filled by connective tissue rich in cells and blood vessels (Fig. 4 B1, B2). Two-way ANOVA not showed differences between treatment in the M% (*p* = 0.3270), CT% (*p* = 0.4341), NB% (*p* = 0.8404), and BM% (*p* = 0.5117) (Fig. 4 C1–C4). However, significant differences were observed among periods in both subgroups (*p* < 0.004). At 60 days, a reduction of 74% in the M% of DBB particles and an increase of 20 and 610% in new bone tissue and bone marrow occurred, respectively. At 180 days, the M% and NB% were similar to period of 60 days, however, the BM% increased 35%, occupying 31.3% of the maxillary sinus (Fig. 4 C1–C4).

**Fig. 5:**
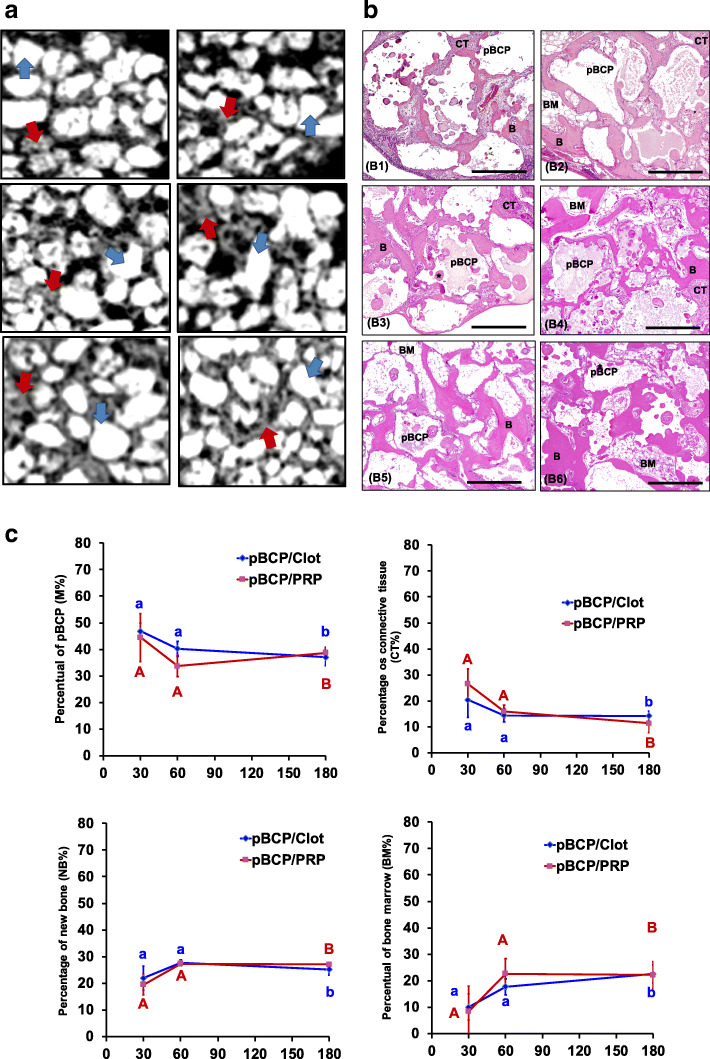
Micro-CT and histomorphometric results for pBCP group. **a** 2D-Micro-CT view: the images show larger and regular pBCP particles (blue arrow) surrounded by new bone (yellow arrow) at 30 days. Between 30 and 180 days, the spaces were filled by bone. **b** Histologic view: at 30 days, the presence of large biomaterial (pBCP) particles (area surrounded by black dotted line) with bone (yellow arrow) formation occurring both on the surface and inside of the small pores and concavities of the particles, and connective tissue (CT) filling the remaining areas. At 60 days, the bone marrow (BM) gradually invaded the spaces between the particles, replacing the connective tissue and being more evident at 180 days. HE; 10×, **c**) Percentage of each constituent: graphs of evolution of M% (**C1**), CT% (**C2**), NB% (**C3**), and BM% (**C4**) in MSA of pBCP/clot and pBCP/PRP. Different letters *p* < 0.05 among periods per group

pBCP group (Fig. 5 and Table 2): although the pBCP particles presented physical differences to the DBB such as, larger size, more regular format, and presence of surface concavities (Fig. 5a, b), the bone regeneration process was similar in both groups (Fig. 4a, b). Two-way ANOVA also not revealed statistical differences between treatment in the M% (*p* = 0.2627), CT% (*p* = 0.6852), NB% (*p* = 0.5200), and BM% (*p* = 0.3325) (Fig. 5 C1–C4). However, significant statistical differences were observed among periods in both subgroups (*p* < 0.0033). Thus, at 30 days, a thin layer of newly formed bone (NB% = 21.9% and 19.5% for pBCP/clot and pBCP/PRP, respectively) was present on the pBCP surface (Fig. 5 B1, B2). Between 30 and 60 days, in both subgroups occurred a small reduction of 83% in the M% (being 46.8% for 40.2% in pBCP/clot and for 44.4% for 35.7% pBCP/PRP Fig. 5 C1). The new formed bone and bone marrow enlarged 30% (from 21.9 to 27.6% in pBCP/clot and 19.5 to 27.3% in pBCP/PRP Fig. 5 C3) and 120% (from 10.1 to 17.7% in pBCP/clot and from 8.5 to 23.5% in pBCP/PRP Fig. 5 C4), respectively, filling most of the spaces between particles previously occupied by connective tissue (CT% of 23.5% at 30 days vs CT% of 14.2% at 60 days, Fig. 5 C2).

### Graft materials (AB; DBB; pBCP) versus treatment (clot; PRP)

**Fig. 6:**
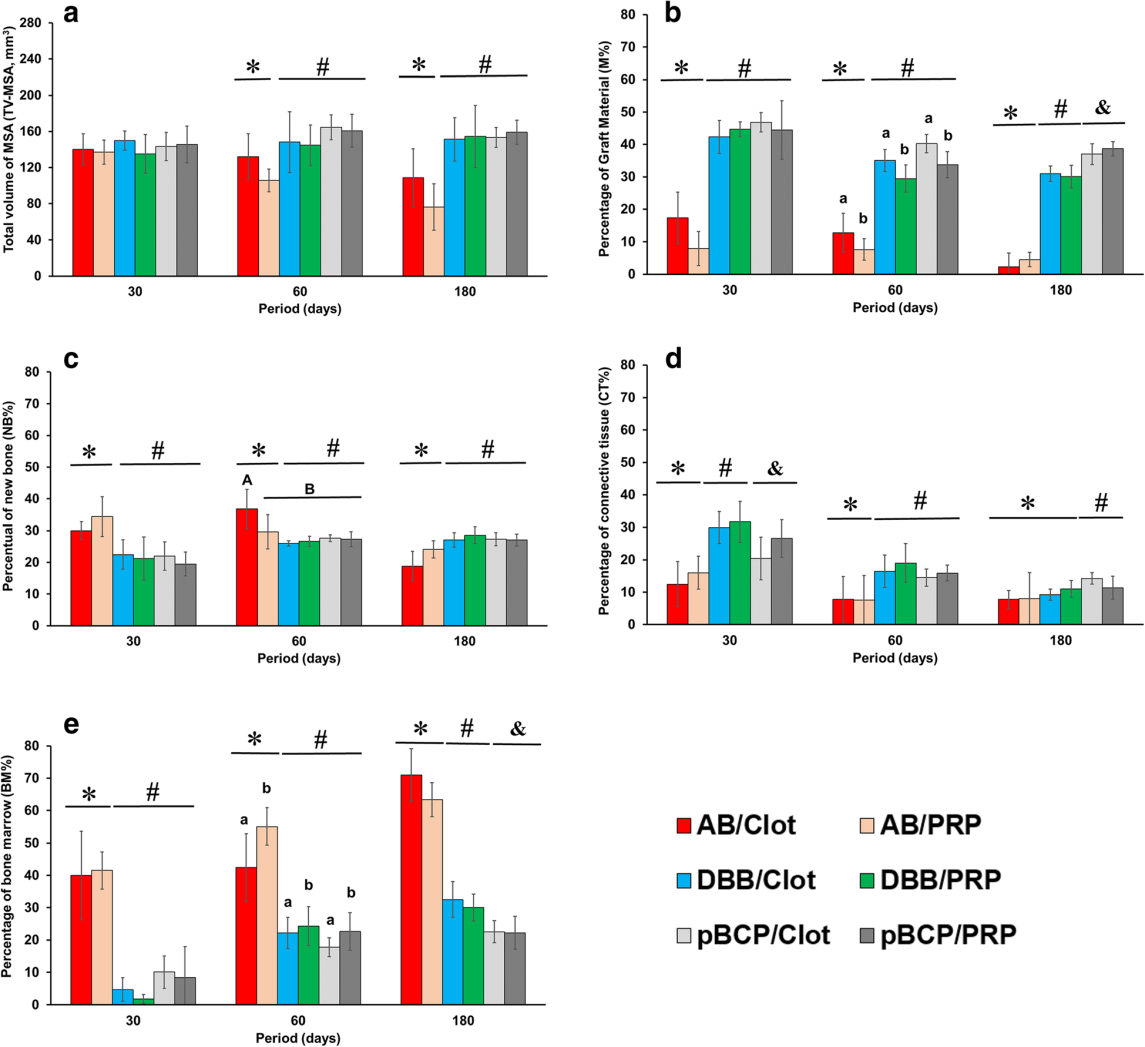
Graft materials versus treatment. Graphs of TV-MSA (**a**), M% (**b**), NB% (**c**), CT% (**d**), and BM% (**e**) obtained by AB/clot, AB/PRP, DBB/clot, DBB/PRP, pBCP/clot, and pBCP/PRP. Two-way ANOVA, different symbols *p* < 0.05 for materials/period; different lowercase letters *p* < 0.05 for treatment/period and different capital letters *p* < 0.05 among groups (interaction between material and treatment)

Two-way ANOVA was used to assess the existence of interaction between the materials and the type of treatment (clot and PRP) (Fig. 6). The TV of MSA at 30 days were similar in all treatment groups (Fig. 6a), with an average of 141.8 ± 16.4 mm^3^. At 60 and 180 days, the TV of MSA showed statistical differences only in relation to the materials (*p* = 0.000001). At 180 days, the TV of MSA in the AB groups were 38% lower than in the DBB and pBCP groups (mean of 150.8 mm^3^) regardless of treatment no differences were observed (*p* = 0.1368).

The percentage of grafted material was also significantly different between groups of materials (*p* = 000000). At 30 days, the percentage of autogenous bone (Fig. 6b) was 72% lower than DBB and pBCP. This difference was maintained throughout the experimental period. At 60 days, M% also showed significant differences between treatment groups (*p* = 0.00086) with lower values for material associated with PRP compared to clot.

Regarding the percentage of new bone (Fig. 6c) and bone marrow (Fig. 6e), significant differences were observed in relation to the type of grafted material (*p* = 0.000037 and *p* = 0.00000, respectively). In the AB groups, the NB% (Fig. 6c) was 35% higher than DBB and pBCP groups at 30 and 60 days, while at 180 days higher values was observed in DBB and pBCPs groups than AB. The rapid formation/remodeling of new bone in AB groups can be seen in the BM% which was 150% higher compared to the DBB and pBCP in all experimental periods. The period of 60 days was critical for observing an interaction between the type of material and the treatment (*p* = 0.045341) with higher NB% in AB/clot compared to the other experimental groups (Fig. 6c). Interestingly, in this period the sinus treated with PRP showed a higher BM% compared to the respective materials associated with the clot (*p* = 009966). The percentage of connective tissue varied in relation to the type of material (*p* < 0.0005) throughout the experimental period. The CT% was lower in sinus filled with AB in relation to DBB and pBCP in the periods of 30 and 60 days, but similar to that of the DBB group at 180 days.

## Discussion

In recent years, PRP has been widely used in several areas of regenerative medicine as a safe and easy option for the treatment of bone, muscle, joint, and skin injuries [19, 20]. Less vascularized areas, such as the maxillary sinus, may benefit from regenerative therapies using PRP since platelets accelerate revascularization in the zone from the injured tissue [21]. The present study evaluated the effect of PRP as an adjunctive material to different bone graft AB, DBB, and pBCP on bone formation after MSA procedure in rabbits. We used a split-mouth design that allow the comparison of PRP and blood-clot associated with the space filler graft materials in the same animal eliminating individual variation in healing process. Although none significative differences occurred in the quantity of new bone between PRP and blood clot during all experimental periods, a higher dynamic in the bone formation, remodeling, and maturation were observed with the use of PRP.

Many factors may influence the function of PRP in bone regeneration [22]. Variations in the obtaining methods and differences in concentration of platelets and growth factors could be associated with the stimulation effect of platelets. There are several protocols for preparation of PRP [16, 22]. In the present study, followed a technique of two centrifugations previously described [16]. The double centrifugation protocol was chosen because the second spin creates a more concentrated and purified PRP [23], with the number of platelet within the range of 1000 to 1845 × 10^3^/μL, which is required for a positive effect on bone regeneration [24]. The total number of platelets in the PRP obtained with this protocol was about 5 times higher than in whole blood (1403 × 10^3^/μL ± 384.1 vs 270 × 10^3^/μL ± 51.8), value that is in agreement with what is defined in the literature as typical PRP [8]. In this context, several studies showed positive effects on bone regeneration when used PRP with similar concentration of platelets [25, 26]. However, in higher concentrations (6–11 times), PRP could have inhibition and cytotoxic effects on osteoblastic activity decreasing cell proliferation, while in lower concentrations (0.5–1.5 times) the effects are suboptimal [27].

Relative to MSA volume, the material plus clot or PRP used in each MSA was of 200 mm^3^. After 30 days post-surgery, the MSA volume reduced similarly in all experimental groups being in mean 142 mm^3^. This reduction occurred due to contraction of the clot/PRP associated with sinus pressure. The MSA volume remained stable in the DBB and pBCP groups until 180 days. However, in the AB groups decreased markedly from 118.8 mm^3^ to 92.5 mm^3^ at 60 and 180 days, respectively, due to the quick and gradual reabsorption of the bone graft particles and new bone remodeling with higher substitution by bone marrow (41%, 49%, and 67% at 30, 60, and 180 days, respectively) as also observed by other authors [28]. It has been shown that the sinus lift with autogenous graft offers excellent bone regeneration, but in the long term, the newly formed bone and the graft particles do not adequately support positive sinus pressure due to respiration, leading to bone resorption [13].

The association of PRP with autogenous bone leads to greater reduction of MSA total volume compared to AB/clot. Comparatively, in AB/PRP, a higher resorption of graft particles than AB/clot was observed at 60 days (M% of 7.6% versus M% of 12.8%, respectively). However, a quick and higher bone formation in AB/PRP (peak at 30 days, NB% = 35%) than AB/clot (peak at 60 days, NB% = 36%) was verified, highlighting that the bone-formation and maturation are accelerated with AB/PRP treatment. It is important to observe that the 60-day period was critical to note the presence of higher %NB in AB/clot compared to the other experimental groups. Comparatively, rabbit calvarial defects treated with autogenous graft and PRP also showed a higher new bone formation at 30 days than at 90 days [25]. This behavior may be related to the fact that the bone tissue has already reached a certain degree of maturation in the later period [29]. A fact that can be corroborated by the higher BM% at 60 days in sinus treated with PRP compared to the respective materials associated with the clot. According to Gerard et al. (2007) [30], PRP could enhance the number of cells recruited including osteoblasts and osteoclasts improving graft resorption and bone formation. Therefore, it is suggested that implant placement in this model could be advanced to a time corresponding to the 30-day period to coincide with the phase of greater bone formation.

Conversely, other preclinical studies did not observe any changes in bone formation associated with treatment with PRP plus autogenous graft [31], while one study verified a delay [32]. However, in human, some studies [9] have reported good results in bone density and quality with the use of autogenous graft with PRP obtained by double centrifugation protocol without platelets quantification.

Both DBB [33, 34] and pBCP [35] are excellent osteconductor material, slowly reabsorbed and that maintains the newly formed bone volume stable even in the long-term period. At 180 days, DBB volume density was 30.5% in this study, similar to that found in biopsies of maxillary sinuses lift in humans collected after 160 days [36], demonstrating the similarity of results among the species. In human biopsies collected after 9 years of sinus lift, the presence of DBB particles intimately adhered to the bone tissue has been observed which proves that DBB replacement is very slow [34]. The present study confirmed that the grafted volume of pBCP remained over 180 days. This is consistent with previous human autopsy study that evaluated the long-term period after sinus lift using BCP 60/40 in humans and also observed successful bone formation and long-term volume stability [37]. Another study demonstrated the stability of BCP with minimal reduction on the height of grafted area 3- to 6 years after sinus lift in humans [38]. Mordenfeld et al. compared BCP and DBB in human maxillary sinus augmentation and observed no statistical differences in grafted area reduction after 5 years [39]. These results are consistent with the obtained in the present study that observed similar results with DBB and BCP treatments.

The lower degradation of DBB and BCP would allow less space for new bone deposition as well as blood vessel formation, comparatively to AB that have higher resorption allowing more space for bone formation. Due to the slow reabsorption and higher osteoconductive properties of pBCP and DBB, newly bone tissue was formed in the spaces between particles and in the pores and concavities of pBCP. The filling of the particle spaces with bone tissue occurred until the period of 60 days, remaining stable up to 180 days.

The association of PRP with DBB or pBCP did not promote differences in the results, both the volume maintenance and amount of new bone tissue formed, were similar. Several studies also did not find statistically significant differences during the bone repair when the PRP was added to both DBB [40] or BCP [41]. The materials associated with blood may have presented similar results to the groups with PRP because the blood also has platelets that despite their lower concentration also release growth factors [40]. Furthermore, according to Roldán et al. (2004), the absence of positive effects in association of PRP with cell-free biomaterials may be due to the lack of osteoinductive capacity of PRP and the absence of osteoblasts in the anorganic bovine bone [42]. Besides, the effects of PRP are deeply related with the type of graft material used, that, just as the surface of the graft material that can alter the form of activation and concentration of the platelets [28].

In summary, it was concluded that all graft materials evaluated here promoted bone and bone marrow formations throughout the evaluated periods. The MSA grafted with AB graft presented a higher and quick bone formation and remodeling than pBCP and DBB. The use of PRP plus AB increased the graft reabsorption and new bone remodeling compared to clot leading to higher reduction of MSA volume at long-term periods. The use of PRP plus DBB or pBCP had no influence on the bone formation/remodeling.

## Data Availability

The datasets used and analyzed during the current study are available from the corresponding author on reasonable request.

## Abbreviations

ANOVA: Analysis of variance
AB: Autogenous bone graft
ß-TCP: Beta-tricalcium phosphate
BCP: Biphasic calcium phosphate
DBB: Deproteinized bovine bone
HE: Hematoxilin and eosin
IGF-1: Insulin growth factor 1
MSA: Maxillary sinus augmentation
NP-B: Total number of platelets in whole blood
NP-PRP: Total number of platelets in PRP
NP-PPP: Total number of platelets in PPP
pBCP: Porous biphasic calcium phosphate
PDGF: Platelet-derived growth factor
PPP: Platelet-poor plasma
PRP: Platelet-rich plasma
TGF-β: Transforming growth factor-beta
TV: Total volume
VEGF: Vascular endothelial growth factor
NB%: Percentage of new bone
M%: Percentage of material
BM%: Percentage of bone marrow
CT%: Percentage of connective tissue
